# Targeting FLT3 in acute myeloid leukemia using ligand-based chimeric antigen receptor-engineered T cells

**DOI:** 10.1186/s13045-018-0603-7

**Published:** 2018-05-02

**Authors:** Ying Wang, Yingxi Xu, Saisai Li, Jia Liu, Yanyan Xing, Haiyan Xing, Zheng Tian, Kejing Tang, Qing Rao, Min Wang, Jianxiang Wang

**Affiliations:** State Key Laboratory of Experimental Hematology, Institute of Hematology and Blood Diseases Hospital, Chinese Academy of Medical Sciences and Peking Union Medical College, 288 Nanjing Road, Tianjin, 300020 China

**Keywords:** Acute myeloid leukemia, Chimeric antigen receptor, FLT3, FLT3-ITD, Immunotherapy

## Abstract

**Background:**

Chimeric antigen receptor-engineered T (CAR-T) cells have extraordinary effect in treating lymphoblastic leukemia. However, treatment of acute myeloid leukemia (AML) using CAR-T cells remains limited to date. Leukemogenesis always relates with the abnormalities of cytogenetics, and nearly one third of AML patients have activating mutations in Fms-like tyrosine kinase 3 (FLT3) which reminded poor prognosis. Considering the FLT3 expressed in AML patients’ blast cells, it may be a new candidate target for CAR-T therapy to treat FLT3^+^ AML, especially patients harboring FLT3-ITD mutation.

**Methods:**

The FLT3L CAR-T using FLT3 ligand as recognizing domain was constructed. The specific cytotoxicity against FLT3^+^ leukemia cell lines, primary AML cells, and normal hematopoietic progenitor stem cells (HPSCs) in vitro were evaluated. In addition, FLT3^+^ AML mouse model was used to assess the effect of FLT3L CAR-T therapy in vivo.

**Results:**

FLT3L CAR-T cells could specifically kill FLT3^+^ leukemia cell lines and AML patients’ bone marrow mononuclear cells in vitro (with or without FLT3 mutation) and have more potent cytotoxicity to FLT3-ITD cells. In a human FLT3^+^ AML xenograft mouse model, FLT3L CAR-T cells could significantly prolong the survival of mice. Furthermore, it was found that FLT3L CAR-T cells could activate the FLT3/ERK signaling pathway of FLT3^+^ leukemia cells with wild-type FLT3; meanwhile, it had no inhibitory effects on the colony formation of CD34^+^ stem cells derived from normal human umbilical cord blood.

**Conclusions:**

The ligand-based FLT3L CAR-T cells could be a promising strategy for FLT3^+^ AML treatment, especially those carried FLT3 mutation.

**Electronic supplementary material:**

The online version of this article (10.1186/s13045-018-0603-7) contains supplementary material, which is available to authorized users.

## Background

Acute myeloid leukemia (AML) remains a disease with a poor clinical prognosis. Although most of patients achieve remission by induction chemotherapy, nearly all of them relapse and need consolidation chemotherapy or hematopoietic stem cell transplantation (HSCT). The majority of patients will ultimately die of their disease, and the 5-year survival rate is only 40–50% [1]. The FMS-like tyrosine kinase-3 (FLT3), a tyrosine kinase receptor, was one of the most frequently mutated genes in AML and accounted for approximately 30% of AML cases [2]. Internal tandem duplication (ITD) mutations of the FLT3 gene occurred in nearly 24% of adult AML patients [3]; the other common mutant type of FLT3 which occurred in the activation loop of the tyrosine kinase domain (TKD), mainly at aspartic acid 835, was found in approximately 7% of AML patients [4]. FLT3-ITD mutations, as well as TKD mutations, led to constitutive activation of FLT3 kinase and triggered several downstream signaling pathways, including the Raf/MEK/ERK pathway, JAK/STAT5 pathway, and PI3K/Akt pathway, which promoted the progression of AML [5]. In general, AML patients with FLT3 mutation represented poor outcomes [6]. Although the development of FLT3 inhibitors might improve the clinical outcome, the only effective treatment for FLT3 mutant AML patients remained allogeneic HSCT currently [7]. Therefore, it is necessary to investigate a new treatment strategy for AML patients with FLT3 mutation.

Chimeric antigen receptor (CAR) T-cell therapy is breaking an innovative new age in treating refractory and recurrent lymphocytic leukemia patients. In particular, CD19-targeted CAR-T cell therapy has achieved dramatic clinical success in treating acute lymphoblastic leukemia (ALL) patients [8]. Up to 90% patients achieved complete remission treating with CD19 CAR-T cells [9]. One major challenge in achieving the amazing clinical success of CAR-T cells in ALL to other types of cancer, including AML, is choosing a proper tumor-associated antigen (TAA) as target. Up to now, several TAAs have been reported as AML antigens against by CAR-T cells such as CD33 and CD123 [10], but previous CAR-T cell therapy trials in AML did not result in long-term responses and exhibited toxicity toward normal hematopoietic progenitor stem cells (HPSCs). Recent study has reported CAR-T cells targeting FLT3 using the single-chain variable fragment (scFv) as recognizing domain of CAR [11], but FLT3 scFv-based CAR-T cells failed to identify wild-type FLT3 from mutated FLT3.

In this study, we tried the ligand-based CAR-T cells to target FLT3. The ligands of FLT3, FLT3L as the recognizing domain and 4-1BB and CD3ζ as the intracellular signal domain, were used to generate a novel FLT3L CAR. T cells were modified to express the FLT3L CAR through a lentivirus system. Then, the functions of CAR-T cells were examined. It showed that FLT3L CAR-T cells were able to robustly kill FLT3^+^ leukemia cells in vitro and showed more potent cytotoxicity toward FLT3-ITD cells than FLT3 wild-type (WT) cells. The downstream signaling pathway of FLT3-WT cells was activated by FLT3L CAR-T, rather than FLT3-ITD cells. The in vivo treatment revealed that FLT3L CAR-T cells could prolong the survival of xenograft AML mice. Moreover, FLT3L CAR-T cells showed a limited toxicity against normal CD34^+^ umbilical cord blood stem cells in vitro. To our knowledge, there was no previous report that ever described such an approach of using ligand-based CAR-transduced effector cells to target the FLT3 molecule in AML treatment.

## Methods

### Cell culture

293T cells were maintained in Dulbecco’s modified Eagle’s medium supplemented with 10% FBS and glutamine. REH, THP-1, MOLM13, and U937 cells were grown in RPMI-1640 with 10% FBS. MV4-11 cells were maintained in Iscove’s modified Dulbecco’s medium supplemented with 15% FBS.

### Identification of *FLT3-ITD* mutations

The multiple mutation domains of *FLT3* gene in exons 14 and 15 were amplified from genomic DNA of cells using the following primers: forward 5′-GCAATTTAGGTAT GAAAGCCAGC-3′ and reverse 5′-CTTTCAGCATTTTGACGGCAACC-3′. A total volume of 50 μl containing 900 ng of genomic DNA was used under the following conditions: denatured at 95 °C for 5 min; annealed at 95 °C for 30 s, 60°C for 30 s, and 72°C for 30 s; and extended at 72 °C for 10 min. The products of PCR were electrophoresed in 3% agarose gels, stained with ethidium bromide, and observed under UV light.

### Construction of FLT3L CAR lentiviral vectors

The FLT3 binding domain of FLT3L [12] (FLT3L-BD) was cloned from the cDNA of a patient’s peripheral blood mononuclear cells (PBMC) by PCR via the following primers: forward 5′-CGCGGATCCACCCAGGACTGCTCCTTCCA-3′ and reverse 5’-CCGGAATTCCTGACACTGCAGCTCCAGGC-3′. The FLT3L-BD was subsequently cloned into pCDH-4-1BB-CD3ζ plasmid which was constructed before [13]. The empty plasmid pCDH was used as control vector.

### Lentivirus production

Recombinant lentivirus was packaged as we previously described [13].

### T cell isolation and infection

The detailed protocol of CD3^+^ T cell isolation has been described previously [13]. Briefly, T cells maintained in X-VIVO15 (LONZA, USA) with 5% FBS, Dynabeads® Human T-Activator CD3/CD28 (Stem Cell, USA), and 50 IU/ml rhIL-2 (R&D, USA) were inoculated in 24-well plates with a cell density of 1 × 10^6^/ml. After 24 h, cells were transduced with FLT3L-CAR lentivirus. Cells transduced with empty plasmid pCDH lentivirus as control (VEC-T). The transduced cells were centrifuged and incubated for another 24 h. The culture medium was changed every other day, and cells were kept in flasks at a density of 3–5 × 10^5^/ml with 50 IU/ml rhIL-2.

### CAR expression and CAR-T cell phenotype analysis

Four days after infection, T cells were harvested and washed once with PBS, stained with rabbit anti-FLT3L antibody (Abcam, USA) for 1 h at 4 °C, and washed twice. Then PE donkey anti-rabbit IgG antibody (Biolegend, USA) was added, incubated at 4 °C for 30 min, and analyzed by flow cytometry using CantoII flow cytometer (BD Biosciences, San Jose, CA, USA) [14]. For T cell phenotype analysis, T cells were harvested 7 days after infection and washed once with PBS, stained with anti-CD4-PE/Cy7 (Biolegend, USA), anti-CD8-PerCP-Cy5.5 (Biolegend, USA), anti-CCR7-PE (Biolegend, USA), and anti-CD45RA-Pacific Blue (Biolegend USA) 30 min at 4 °C, then washed and resuspended in PBS for flow cytometry analysis [15].

### CAR-T specific killing assay

#### CART-T specific killing assay for cell lines

FLT3L CAR-T (or VEC-T) cells and target cells were co-cultured in a 24-well plate with an E:T ratio of 1:8, 1:4, 1:2, and 1:1 in 1 ml medium (X-VIVO15 with 5% FBS) for 48 h. Cells were harvested and washed once, stained with anti-CD3-APC/Cy7 (Biolegend, USA) and anti-CD19-APC (REH cells) or anti-CD33-APC (THP-1, MOLM13, MV4-11 and U937 cells) for 30 min at 4 °C, then washed and resuspended in PBS for flow cytometry analysis. The percentage of CD19^+^ cells (REH) or CD33^+^ cells (THP-1, MOLM13, MV4-11 and U937) represented the residual level of target cells.

#### CART-T specific killing assay for primary AML cells

Bone marrow mononuclear cells (BMMCs) containing 44~ 98% AML blasts were isolated from bone marrow aspirates of AML patients through Ficoll-Paque density centrifugation and frozen in liquid nitrogen until use. All the samples obtained from the patients enrolled in the Institute of Hematology and Blood Diseases Hospital were collected under informed consent.

FLT3L CAR-T (or VEC-T) cells and primary AML cells were co-cultured in a 24-well plate with an E:T ratio of 1:4 in 1 ml medium (X-VIVO15 with 5% FBS) for 48 h. The followed staining steps were performed as mentioned above. AML cells were detected using anti-CD33-APC antibody. The percentage of CD33^+^ cells represented the residual level of target cells.

### Degranulation assay

FLT3L CAR-T (or VEC-T) cells and target cells were co-cultured in a 96-well plate with an E:T ratio of 1:1 in triplicate in 200 μl medium with anti-CD107a-PE antibody and monensin (Sigma, USA). After 4 h, cells were labeled with anti-CD3-APC/Cy7 antibody and analyzed by flow cytometry.

### Cytokine release assay

FLT3L CAR-T (or VEC-T) cells and target cells were co-cultured in a 24-well plate with an E:T ratio of 1:1 in triplicate in 1 ml medium for 24 h. Supernatants were harvested and assayed for the production of IFN-γ, TNF-α, and IL-2 by ELISA (R&D, USA), and the absorbance at 450 nm was measured using Synergy H4 Hybrid Microplate Reader (Biotek, Winooski, VT, USA).

### Colony formation assay

CD34^+^ cells from cord blood were sorted after labeling with CD34 magnetic beads (Miltenyi Biotec, Germany) according to manufacturer’s instructions. Viability and purity of the sorted CD34^+^ cells were determined by flow cytometry. CD34^+^ cells were co-cultured with CAR-T or VEC-T cells for 24 h, and then, 500 CD34^+^ cells from each group were seeded in a methylcellulose medium (H4434, Stem Cell, Canada) and incubated at 37 °C, 5% CO_2_. After being cultured for 14 days, colonies (determined as 100 cell/colony) were scored and recorded by Nikon Eclipse Ti-U fluorescent microscopy under a × 10 objective (Nikon, Japan).

### Intracellular phosphorylated protein staining assay

FLT3L CAR-T (or VEC-T) cells and target cells were co-cultured in a 24-well plate with an E:T ratio of 1:8 in triplicate in 1 ml medium for 4 h. Cells were fixed by adding an equal volume of IC Fixation Buffer (eBioscience, USA) and vortexed to mix. Cells were incubated for 1 h at room temperature (RT), protected from light, and then, cells were washed and fixed by using 1 ml ice-cold methanol. Cells were vortexed to mix and incubated for 30 min at 4 °C. Cells were washed twice in PBS then resuspended in 300 μl PBS within 1 μl intracellular phospho-epitope antibody (anti-p-STAT5-PE, anti-p-ERK-PE, or anti-p-AKT-PE, eBioscience, USA) and 1 μl anti-CD33-APC for 30 min at RT. Washed cells were resuspended in PBS and analyzed by flow cytometry.

### In vivo NOD/SCID murine studies

Female NOD/SCID mice about 6 to 8 weeks old were obtained from Institute of Laboratory Animal Sciences (CAMS&PUMC, China). After sublethal irradiation, 14 mice were inoculated with 5 × 10^6^ MV4-11 cells intravenously. Seven days after inoculation, mice were randomized into two treatment groups. Group CAR-T and group VEC-T were respectively injected intravenously with 1 × 10^7^ CAR-T cells or VEC-T cells at day 7 and day 14. Body weight and the overall survival of mice were recorded. Dead mice were dissected for pathological and flow cytometry analysis to confirm the diagnosis of leukemia. All animal experiments were approved by the Institutional Animal Care and Use Committee of Peking Union Medical College.

### Statistical analysis

Values were expressed as mean ± SD. Significance were determined by Student’s *t* test using GraphPad Prism (version 5.0). The survival of mice was analyzed by Kaplan-Meier methods and a log-rank test. *p* values < 0.05 were considered statistically significant. *p < 0.05, **p < 0.01, and ***p < 0.001 in comparison.

## Results

### Generation of anti-FLT3 CAR

**Fig. 1:**
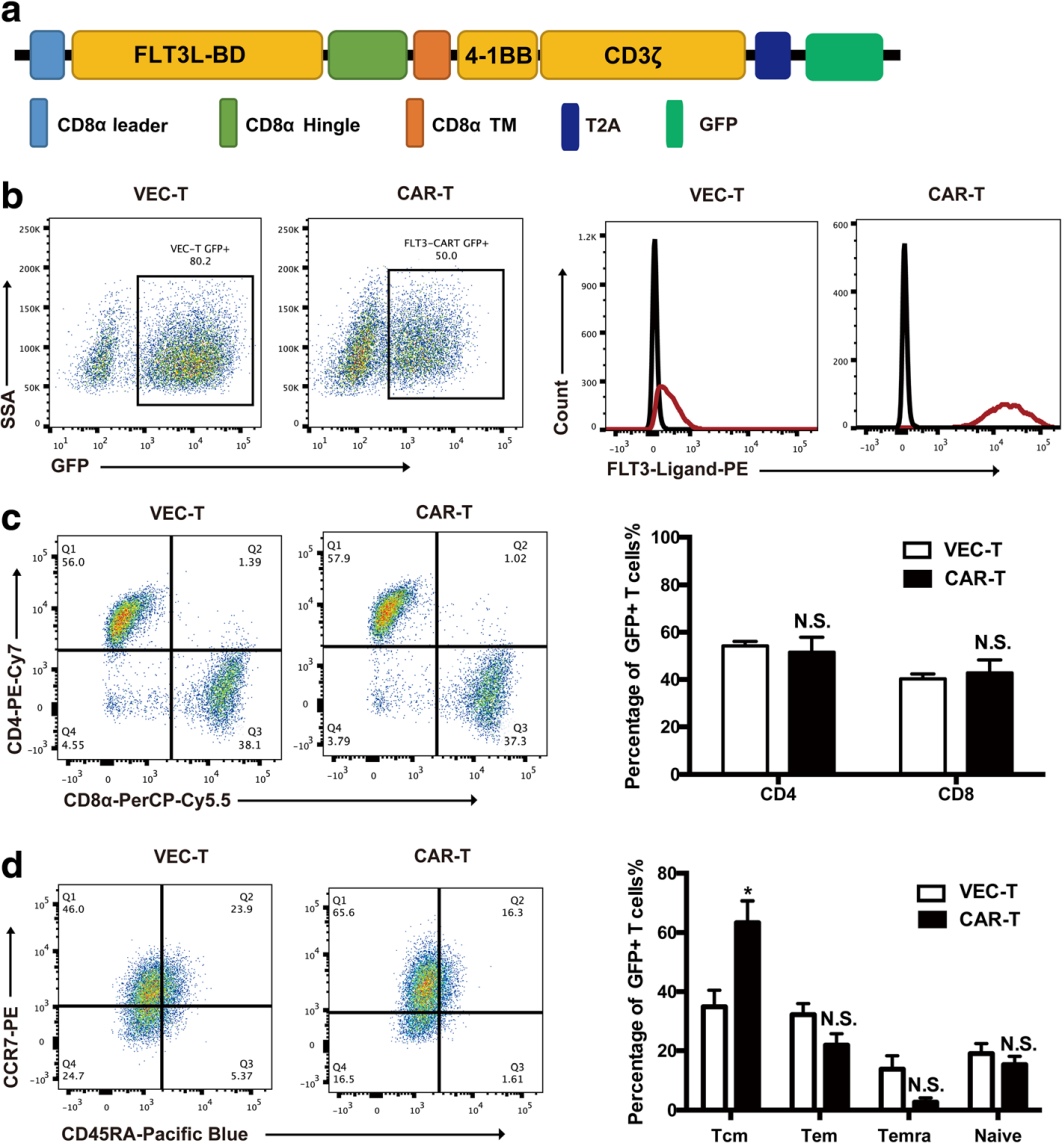
Generation of anti-FLT3 CAR. **a** Schematic diagram of anti-FLT3 CAR structure containing CD8α leader, FLT3L-BD, CD8α hinge and transmembrane domains, and intracellular tandem with 4-1BB and CD3ζ signaling domain; GFP was separated with T2A. **b** Four days after lentiviral transduction, GFP and FLT3L expression on FLT3L CAR-T cells was measured using flow cytometry. **c** The ratio of CD4^+^ and CD8^+^ T cells in CAR-T or VEC-T cells was detected using flow cytometry. **d** CCR7 and CD45RA surface staining was performed on both CAR-T and VEC-T cells. Tcm (central memory, CCR7^+^CD45RA^−^), Tem (effector memory, CCR7^−^CD45RA^−^), Temra (terminally differentiated effector memory, CCR7^−^CD45RA^+^)

The FLT3L binding domain (BD) was cloned into previously verified lentiviral constructs [13], containing CD8α hinge and transmembrane domains, and intracellular tandem with 4-1BB and CD3ζ signaling domain, referred to as FLT3L CAR. To identify transduced T cells, GFP was included in the construct and separated with FLT3L CAR by a T2A peptide (Fig. 1a). Transduction efficiencies of human T cells with FLT3L CAR construct were about 40 to 50% and repeatable as measured by GFP; all GFP-positive FLT3L CAR-T cells expressed FLT-3 ligand which were detected by using anti-FLT3L antibody (Fig. 1b). After expansion for 14 days, CAR expression was maintained in both CD4^+^ and CD8^+^ T cells with a usual CD4:CD8 ratio of 60:40 (Fig. 1c). According to the expression of CD45RA and CCR7 surface markers, which were used to determine the distribution of T cell subpopulations (Tcm, Tem, Temra, and native T cells), it was demonstrated that CAR-T cells had a higher percentage of CCR7^+^CD45RA^−^ (Tcm); however, no significant difference was noted between CAR-T or VEC-T cells in Tem and Temra (Fig. 1d).

#### FLT3L CAR-T cells exhibited antigen-specific cytotoxicity against FLT3^+^ leukemia cells

**Fig. 2:**
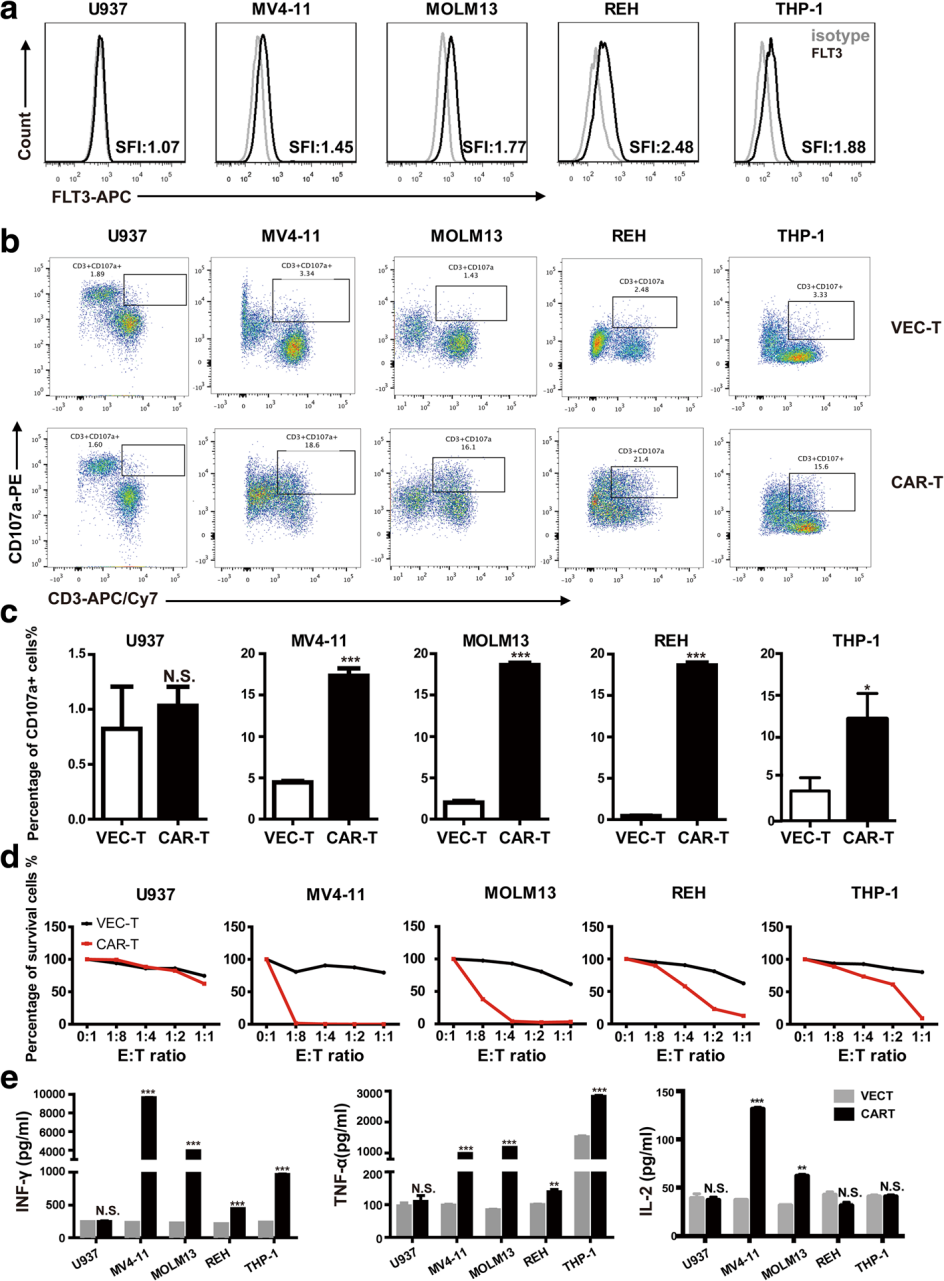
FLT3L CAR-T cells exhibited antigen-specific cytotoxicity against FLT3^+^ leukemia cells. **a** FLT3 expression in AML cell lines analyzed by flow cytometry after staining with anti-FLT3 antibody (black line); the corresponding isotype (gray line) is used as negative control. SFI of FLT3 expression was calculated by dividing the median fluorescence of FLT3 mAb staining by the median fluorescence of mouse IgG1 κ isotype control. **b**, **c** Percentage of CD3^+^CD107a^+^ T cells after being co-cultured with target cells for 5 h was determined by flow cytometry. **d** Cytotoxic activity of FLT3L CAR-T against FLT3-positive or FLT3-negative AML cell lines. Target cells and effector cells were co-cultured for 48 h at the indicated E:T ratio. Anti-CD33 or anti-CD19 antibody and anti-CD3 antibody were used to recognize different cell types. **e** CAR-T or VEC-T cells were co-cultured with leukemia cells for 24 h. IFN-γ, TNF-α, and IL-2 amounts in the supernatants were analyzed by using ELISA

To evaluate the specific cytotoxicity of FLT3L CAR-T cells, FLT3 expression on several AML cell lines was analyzed by specific fluorescence indices (SFI) using flow cytometry and FLT3-positive (FLT3^+^) leukemia cell lines MV4-11 (SFI:1.45), MOML13 (SFI:1.77), REH (SFI:2.48), and THP-1 (SFI:1.88) were used as target cells, FLT3-negative (FLT3^**−**^) cell line U937 (SFI:1.07) was used as control target cells, and VEC-T cells were used as control effector cells (Fig. 2a). To detect T cell cytolytic function, the expression of CD107a surface marker was measured using T cell degranulation assay [16]. After being co-cultured for 5 h, the higher expressions of CD107a were observed in FLT3L CAR-T cells co-cultured with FLT3^+^ leukemia cells (Fig. 2b, c). To further validate the cytolytic capability, FLT3L CAR-T cells and leukemia cells were co-cultured at the indicated effector/target (E:T) ratios for 48 h. As showed in Fig. 2d, even in the lowest E:T ratio of 1:8, MV4-11 cells could not survive and only 30% of MOLM13 cells could survive. If the E:T ratio increased to 1:4, no MOLM13 cells could survive. However, REH and THP-1 cells could survive up to the highest E:T ratio of 1:1. As to the FLT3^−^ cell line U937, the cytolytic activity was not observed. Therefore, compared with FLT3 wild-type cell lines REH and THP-1 (Fig. 5a), FLT3L CAR-T cells showed stronger cytolytic effect on FLT3 mutated (FLT3-ITD) cell lines MV4-11 and MOML13 cells (Fig. 5a). Cytokine release in the co-culture supernatant was assayed to evaluate the cytotoxicity efficacy of CAR-T cells against target cells by ELISA. When CAR-T cells are co-cultured with FLT3^+^ leukemia cells, the levels of interferon-γ (IFN-γ) and tumor necrosis factor-α (TNF-α) were significantly increased compared with those co-cultured with VEC-T cells, while the cytokines could not be detected in CAR-T co-cultured U937 cells, the FLT3^−^ cells (Fig. 2e). However, interleukin-2 (IL-2) expression increased slightly in FLT3-ITD and CAR-T system and showed no difference in FLT3-WT group (Fig. 2e). As it is similar with the data above, CAR-T cells secreted more proinflammatory cytokines when co-cultured with MV4-11 and MOML13 than that with REH and THP-1.

#### FLT3L CAR-T cells showed specific cytotoxic capability against FLT3^+^ primary AML

**Fig. 3:**
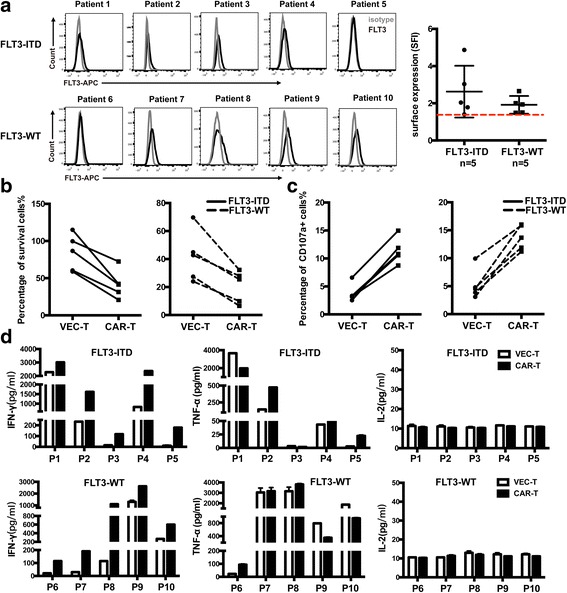
FLT3L CAR-T cells showed specific cytotoxic ability against FLT3 positive primary AML. **a** FLT3 surface expression on patients’ AML cells (44~ 95% blast count; patients 1 to 5, FLT3-ITD; patients 6 to 10, FLT3 wild type) was investigated by flow cytometry. Black line, anti-FLT3; gray line, isotype control. SFI of FLT3 staining was calculated by dividing median fluorescence obtained with the FLT3 mAb by median fluorescence obtained with the mouse IgG1 κ isotype control. The red dotted line represented SFI 1.4 as a defined threshold for FLT3 positivity. **b** Cytotoxic activity of FLT3L CAR-T against FLT3-ITD (bold line) or FLT3-WT (dotted line) primary AML cells. Target cells and effector cells were co-cultured for 48 h at the indicated E:T ratio of 1:4. Anti-CD33 antibody and anti-CD3 antibody were used to recognize different cell types. **c** Percentage of CD3^+^CD107a^+^ T cells after being co-cultured with FLT3-ITD (bold line) or FLT3-WT (dotted line) primary AML cells for 5 h was determined by flow cytometry. **d** CAR-T or VEC-T cells were co-cultured with primary AML cells for 48 h. IFN-γ, TNF-α, and IL-2 amounts in the supernatants were detected by using ELISA

To verify the cytotoxicity efficacy of FLT3L CAR-T cells to primary AML cells, we co-cultured FLT3L CAR-T cells with primary AML cells from 10 patients **(**Table 1**)**, including 5 FLT3-ITD patients (nos. 1–5) and 5 FLT3-WT patients (nos. 6–10). FLT3 expression on primary AML cells was analyzed by flow cytometry; the specific fluorescence indices (SFI) > 1.4 is defined as FLT3 positive **(**Fig. 3a and Table 1**)**. The significant specific cytotoxicity (Fig. 3b), high expression of CD107a (Fig. 3c), and IFN-γ release (Fig. 3d) were observed in FLT3L CAR-T cells co-cultured with both FLT3-ITD and FLT3-WT patient leukemia cells compared to that in VEC-T cells co-cultured with patient cells, while secretion of IL-2 showed no difference, similar to the results of cell lines (Fig. 3d). Some patient samples showed high TNF-α secretion co-cultured with VEC-T cells, which might be due to the severe unspecific cytotoxicity caused by major histocompatibility complex (MHC) incompatibility (Fig. 3d). The results suggested that FLT3L CAR-T cells were capable of recognizing FLT3^+^ (FLT3-WT and FLT3-ITD) primary AML cells and displayed cytotoxicity efficacy.

**Table 1:** Patient information

Patient ID	Age	Sex	Cytogenetics	CD33 (%)	FLT3 (SFI)
1	46	F	FLT3-ITD NPM1 p.W288fs (Exon12) DNMT3A p.r882C (Exon23) PTPN11 p.G60R (Exon3)	90.5	3.03
2	45	M	FLT3-ITD	95.8	2.03
3	34	F	FLT3-ITD PML-RARα	97	1.78
4	52	F	FLT3-ITD NMP1 p.W288fs	96.32	4.87
5	45	F	FLT3-ITD PML-RARα	94.1	1.40
6	46	F	FLT3-WT DNMT3A p.R882C (Exon23)	65.5	1.49
7	27	M	FLT3-WT CBFβ-MYH11 NRAS p.G12D (Exon2) NRAS p.Q61K (Exon3)	91.2	1.94
8	26	F	FLT3-WT CBFβ-MYH11 NRAS p.Q61K (Exon3)	44	1.5
9	19	F	FLT3-WT CBFβ-MYH11 KIT p.N822K (Exon17) NRAS p.G13D (Exon2)	66	2.65
10	34	F	FLT3-WT CBFβ-MYH11 NRAS p.Q61H (Exon3)	89.6	2

*F* female, *M* male, *SFI* specific fluorescence indices

#### FLT3L CAR-T cells prolonged the survival of AML mice

**Fig. 4:**
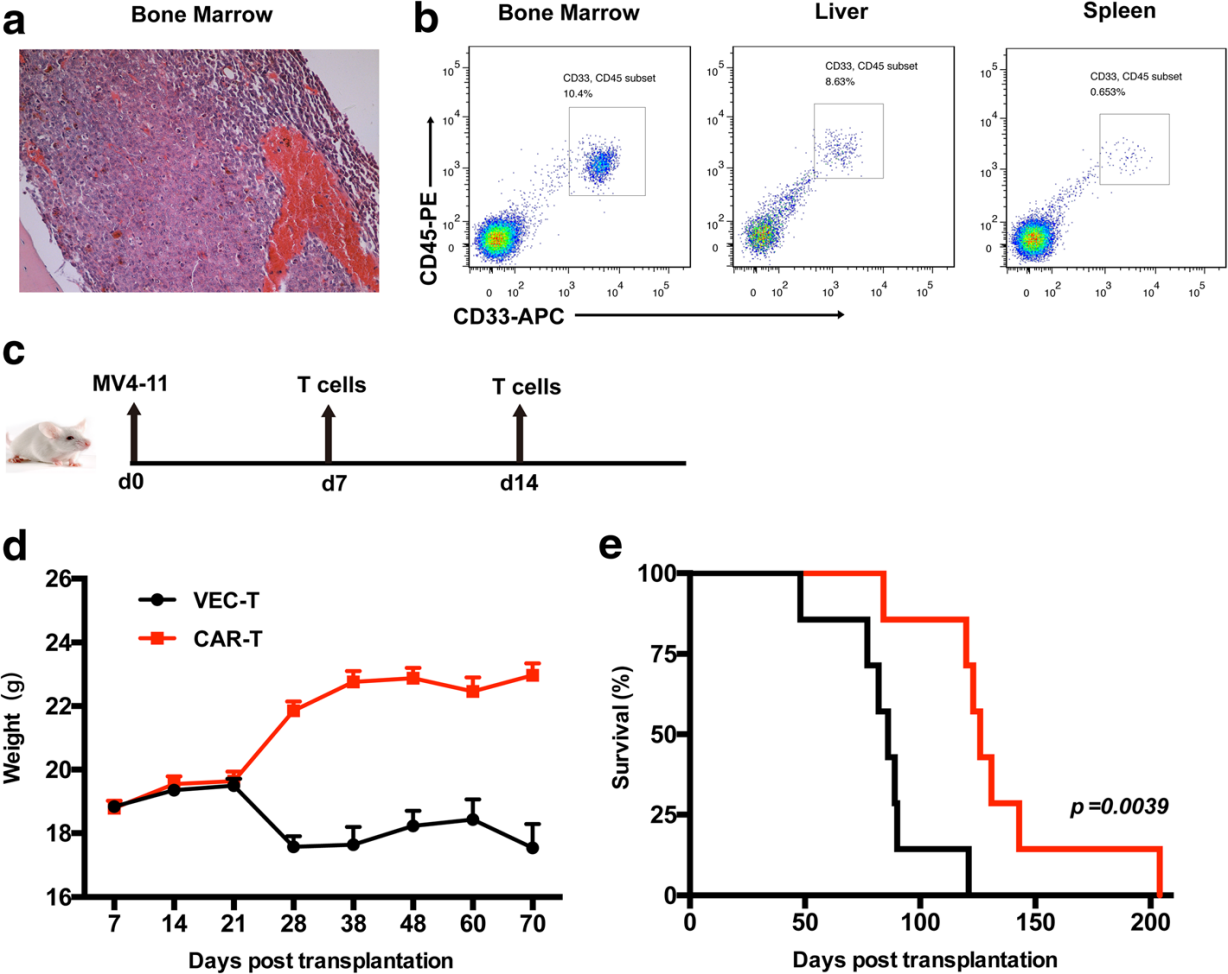
FLT3L CAR-T cells prolonged the survival of AML mice. **a**, **b** FLT3^+^ AML NOD/SCID murine model. Histopathologic analysis of bone marrow and flow cytometry analysis of bone marrow, liver, and spleen showed intensive infiltration of leukemic cells. **c** MV4-11 cells (5 × 10^6^ per mouse) were injected into mice intravenously, 7 and 14 days after leukemia cells inoculation, and 1 × 10^7^ CAR-T cells or VEC-T cells were injected intravenously, *n* = 7 mice per group. **d** Weight change from the day MV4-11 was injected is shown. **e** Kaplan-Meier survival curves for mice with MV4-11, treated with CAR-T or VEC-T cells. *p* = 0.0039, as determined by log-ranked test

To evaluate the in vivo function of FLT3L CAR-T cells, the xenograft FLT3^+^ AML model was established by intravenously inoculating MV4-11 cells into NOD/SCID mice. CD45^+^CD33^+^ cells (leukemia blasts) in peripheral blood could be detected at the end stage of leukemia development (Additional file 1: Figure S1) and extensive infiltrations of AML cells could be detected in bone marrow, liver, and spleen confirmed by pathology (Fig. 4a) and flow cytometry (Fig. 4b). At days 7 and 14 after transplantation, CAR-T and VEC-T cells were intravenously administered (Fig. 4c). Body weight of both CAR-T and VEC-T treated mice showed no significant decrease, indicating little toxicity of CAR-T therapy. Until 28 days after transplantation, body weight of VEC-T group started to drop due to the progress of disease (Fig. 4d). The median survival time of CAR-T treatment group (126 days) prolonged significantly compared to that of VEC-T treatment group (86 days) (Fig. 4e). In brief, CAR-T-treated mice showed a longer survival time compared to that of VEC-T treated mice (*p* = 0.0039).

#### FLT3L CAR-T promoted the phosphorylation of ERK1/2 in FLT3-WT leukemia cells

**Fig. 5:**
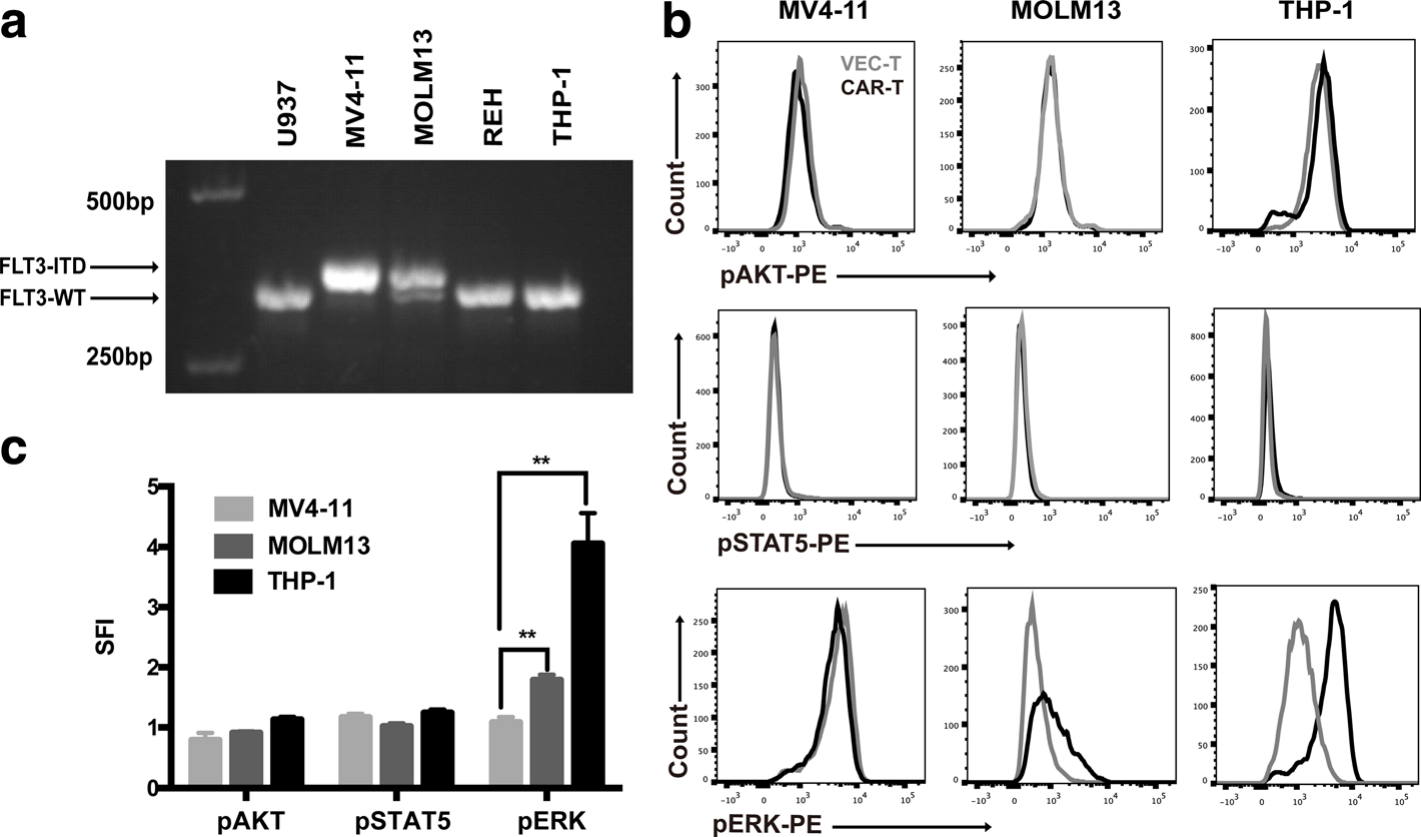
Effect of FLT3L CAR-T on STAT5, AKT, and ERK1/2 phosphorylation of FLT3^+^ leukemia cells. **a** FLT3 mutation types were assessed by PCR, using primers located in 14 and 15 exons as described in the “Methods” section. The upper arrow indicated the FLT3/ITD band, while the lower arrow pointed to the normal FLT3 band. **b** The fluorescence intensities of pSTAT5, pAKT, and pERK1/2 in CD33^+^leukemia cells were measured by intracellular staining with corresponding antibody and analyzed by using flow cytometry. Black histograms indicated fluorescence intensity of leukemia cells in the CAR-T group, while gray histograms indicated that of the VEC-T group. **c** SFI levels of pSTAT5, pAKT, and pERK1/2 obtained through dividing median fluorescence of leukemia cells in CAR-T group by median fluorescence of leukemia cells in VEC-T group

As stated above, it was interesting that FLT3L CAR-T cells showed greater cytotoxic capability against MV4-11 and MOML13 cells than that against REH and THP-1 cells (Fig. 2d). Therefore, we investigated why FLT3^+^ leukemia cells responded differently to FLT3L CAR-T cells. The *FLT3* genotypes of MV4-11, MOLM13, REH, and THP-1 cells were firstly identified, and the results showed that MV4-11 cells expressed the homozygous FLT3-ITD mutation, MOLM13 cells displayed the heterozygous FLT3 mutation (ITD/WT), and REH and THP-1 expressed the normal FLT3 (WT/WT) (Fig. 5a). It is generally considered that ITD mutation led to ligand-independent activation and resulted in the phosphorylation of the downstream signaling pathway of FLT3. On the contrary, the signaling activation of FLT3-WT was ligand-dependent [17]. Therefore, different genotypes of *FLT3* might have distinct response to FLT3L [18]. So, the phosphorylation of STAT5, AKT, and ERK1/2 which might be activated by FLT3L in FLT3^+^ leukemia cells and promote the survival of leukemia cells was further explored. As measured by intracellular phospho-protein staining, the SFI [19] levels of pERK1/2 was significantly higher in FLT3-WT cells (THP-1) than in FLT3-ITD cells (MV4-11, MOLM13) and higher in heterozygous ITD cell cells (MOLM13) than in homozygous ITD cells (MV4-11) (Fig. 5b, c). These data suggested that the antigen-recognizing domain of CAR, FLT3L, activated the FLT3 downstream signaling pathway in the cells with FLT3-WT which demonstrated that FLT3L CAR-T had the ability to distinguish between FLT3 wild-type cells and FLT3-ITD mutant cells and might have little cytotoxicity on cells with normal *FLT3* genotype.

#### FLT3L CAR-T cells did not inhibit CD34^+^ colony formation.

**Fig. 6:**
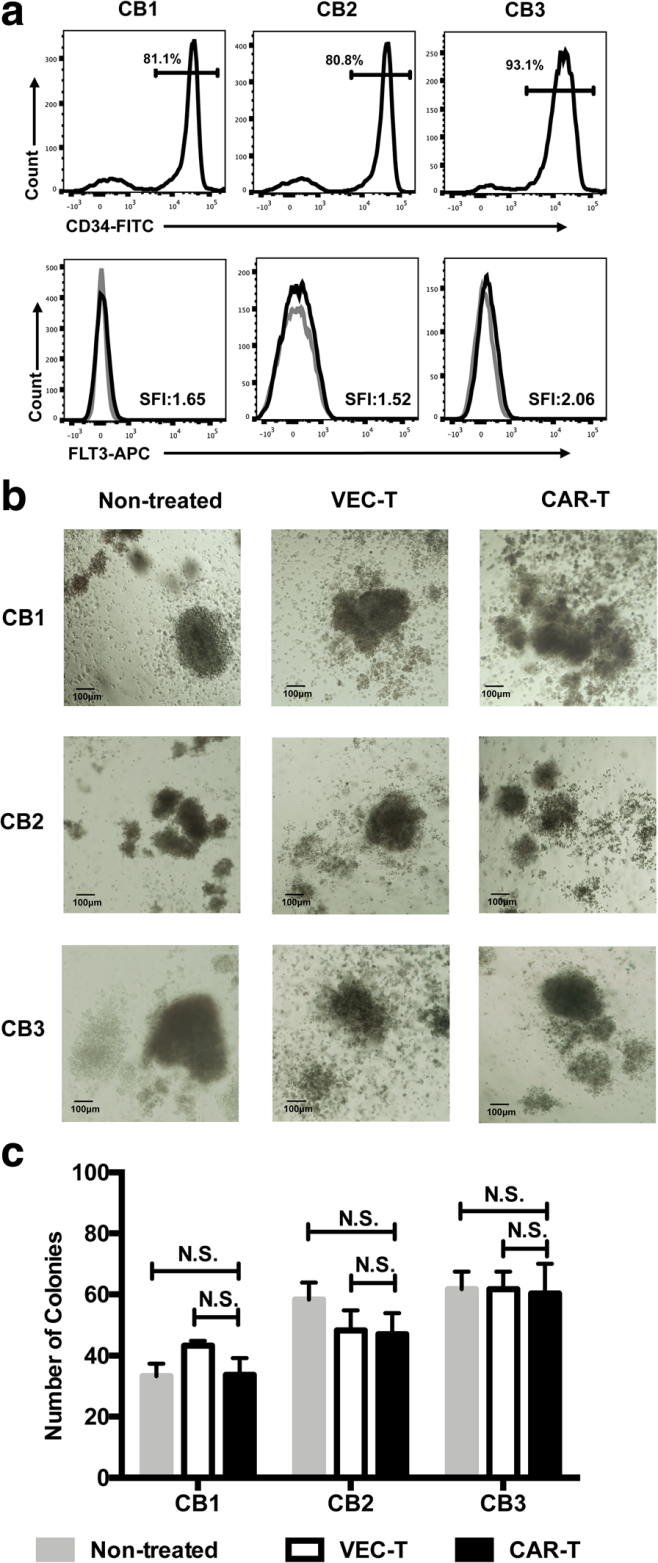
FLT3L CAR T cells did not inhibit CD34^+^ cell colony formation. **a** The purity of CD34^+^ cells isolated from cord blood using the CD34 MicroBead Kit and expression of FLT3 on CD34^+^ cells were measured by flow cytometry. **b** Morphology of colonies (inverted microscope, × 10). CD34^+^ cells of cord blood (500 per well, from three different cord blood samples) co-cultured with VEC-T or CAR-T for 24 h, then treated or untreated CD34^+^ cells incubated in methylcellulose medium for 14 days. **c** Colony numbers of CD34+ cells per well

Many surface markers used as targets to treat AML by CAR-T cells, such as CD33 and CD123, existed in both AML blasts and HPSCs. It had to be considered the on-target/off-tumor effect of CAR-T therapies which was the potential of depletion of normal HPSCs [20]. FLT3 is reported to be expressed on HPSCs and granulocyte/macrophage progenitor (GMP) stages and performs important roles in maintaining the survival of stem and progenitor cells [21]. To investigate whether FLT3L CAR-T cells had cytotoxicity against hematopoietic progenitors, colony formation assay of pre-treated CD34^+^ HSCs derived from umbilical cord blood with CAR-T cells was performed [22]. The purity of the sorted CD34^+^ cells and their expression of FLT3 were measured by flow cytometry, and the FLT3 SFI of the three samples were 1.65, 1.52, and 2.06, respectively (Fig. 6a). It showed that FLT3L CAR-T cells did not inhibit colony formation of normal CD34^+^ cells (Fig. 6b, c). Taken together, our findings suggest that FLT3 can be safely pursued as a target for ligand-based CAR T cell therapy of AML without harming essential healthy HPSCs.

## Discussion

AML patients with favorable prognostic factors responded well to conventional chemotherapy which combined with cytarabine and anthracycline compounds. However, patients with adverse prognostic factors inevitably relapse and have poor prognosis after chemotherapy. To achieve better or even optimal treatment response and reduce treatment-related mortality, strategies such as immunotherapy have been applied to the treatment of hematologic malignancies [23].

Over the past decade, the novel immunotherapy strategy CAR-T cells have revolutionized leukemia treatment, especially CD19 CAR-T. Chimeric antigen receptor (CAR) is an artificial structure including antigen-binding domain which could recognize TAA, transmembrane region, and signal transduction region which could activate T cell to kill tumor cells. A large amount of studies have shown that CD19 CAR-T had striking clinical responses in relapsed ALL and CLL [24]. Meanwhile, researchers are trying to treat AML patients with CAR-T therapy and several AML CAR-T cell therapies targeting relatively specific AML-associated surface markers are under clinical investigation [24, 25]. Up to now, potential target antigens for AML treatment by CAR-T cells were under investigation, including CD33 [26], CD123 [22], CD38 [27], CD44v6 [28], FRβ [29], PR1/HLA-A2 [30], CLL-1 [31], and LeY [32]. CD33 and CD123 are highly expressed on primary AML cells and are the most commonly used antigens for AML treatment by CAR-T therapy [22, 33]. However, these antigens did not only restrictively express on AML cells, they also expressed in various degrees on normal HSPCs, immune cells, and normal healthy tissues. Therefore, looking for AML-associated surface antigens which are specifically expressed on leukemia cells (or even leukemia stem cells), but are low or not expressed on normal cells and tissues, is an ideal strategy to develop CAR-T clinical application [25]. Besides, hematopoietic malignancies always arise with cytogenetic alterations, so the choice of AML-related target antigens involved in leukemogenesis may be a feasible approach for AML CAR-T therapy.

Due to the high expression of FLT3 in 70 to 100% of AML patients and in a high percentage of ALL cases [3], and approximately 30% of AML patients carried the FLT3 mutation which confers proliferative and survival advantages for leukemia cells, FLT3 may become the potential and ideal target for generating CAR-T cells and possess even more effective cytotoxicity on FLT3-ITD AML. The scFv-based FLT3 CAR-T therapy had been developed and demonstrated its potential on the treatment of FLT3 AML. Although it had been reported that FLT3 CAR-T cells did not deplete CD34^+^ HSPCs and preserved HSPC differentiation [11], the scFv-based FLT3 CAR-T cells had similar cytotoxic effect on the cells with FLT3-WT and mutant FLT3 phenotypes.

In our study, a novel type of CAR to target FLT3 using its ligand as recognizing domain, the FLT3L CAR, was constructed. FLT3L CAR-T could interact and eradicate both FLT3^+^ leukemia cell lines and primary AML cells at the E:T ratio as low as 1:8. Nevertheless, the effect of specific cytolysis seemed independent of FLT3 expression level on leukemia cells but relied on whether they carried FLT3-ITD mutation, and the FLT3-ITD-positive leukemia cells were more sensitive to FLT3L-based CAR-T cells than the FLT3 wild-type leukemia cells. Since FLT3 ligand promotes the dimerization and phosphorylation of FLT3 within 5–15 min and subsequently activates several pathways involved in cell survival and proliferation, such as RAS/RAF/Erk, PI3K/Akt/mTOR, and JAK/STAT [34], it was supposed that FLT3L expressed by CAR-T cells might promote dimerization and activation of FLT3 in FLT3-WT cells, and activation of the signal transduction cascades in FLT3 wild-type leukemia cells may promote their survival and relieve damage. To address this question, we measured the phosphorylation level of STAT5, AKT, and ERK in FLT3^+^ leukemia cells after being co-cultured with CAR-T or VEC-T cells and found the increased activation of pERK in FLT3-WT and heterozygous FLT3-ITD leukemia cells, which indicated that the expression of FLT3L on CAR-T cells is involved in activating downstream proliferative Ras-Raf-Mek-Erk pathways. As a result, proliferation and survival of FLT3-WT leukemia cells were promoted. In other words, this may be the reason why the FLT3 wild-type leukemia cells were insensitive to FLT3L-based CAR-T cells. Besides that, FLT3L CAR-T may show comparative stronger cytotoxic capability on leukemia cells harboring FLT3-ITD mutation which is reflecting a very poor prognosis.

TAAs which are restricted to express on tumor cells are ideal target candidates for CAR-T therapy. Unfortunately, antigens used in previous CAR-T studies are also expressed on normal cells and tissues. In clinical application, the on-target/off-tumor toxicities may arise during CAR-T therapy. Rosenberg et al. reported that T cells transduced with a CAR-recognizing ERBB2 could also recognize low levels of ERBB2 on lung epithelial cells, which caused patient’s serious respiratory distress and cytokine storm, eventually leading to the death of the patient [35]. Maude et al. also reported that some CD19 CAR-T treated patients suffered from clinically tolerable B-lymphocyte aplasia for months to years [36]. However, AML CAR-T therapy may induce severe on-target/off-tumor myeloablation and may even threaten life [37]. Kenderian et al. [38], Gill et al. [39], and Wang et al. [40] have reported that CD33 CAR-T treatment could impair normal hematopoiesis. As early myeloid and lymphoid progenitor cells also express FLT3, we detected the FLT3 expression on CD34^+^ HSPCs, which showed no significant difference compared with five leukemia cell lines and leukemia cells of ten AML patients using SFI as an index (Additional file 1: Figure S2). Therefore, CAR-T cell that recognizes FLT3 may induce on-target/off-tumor toxicities. scFv-based FLT3 CAR-T [11] showed no cytotoxicity to PBMCs of healthy donors, and CD34^+^ HSCs isolated from cord blood in vitro and in vivo. To test whether FLT3L CAR-T could be more safe, colony formation assay of pre-treated CD34^+^ HSCs derived from umbilical cord blood with CAR-T cells was performed in our study. It showed that FLT3L CAR-T cells did not inhibit colony formation of normal CD34^+^ cells. It was reported that FLT3 and FLT3L combination and activation of the downstream pathway had a pivotal role in the regulation of hematopoietic cells, including phospholipid metabolism, transcription, proliferation, and apoptosis [34]. Based on our results described above, FLT3L CAR-T has the potential to activate survival-associated signaling pathways of CD34^+^ HSPCs expressing FLT3-WT and reduce cytotoxicity to some extent, which is also indicating the possible safe use of FLT3L CAR-T. However, clinical application of FLT3L CAR-T requires more evidences to verify its on-target/off-tumor toxicity.

Proliferation and activation of CAR-T cells in vitro or in vivo occurred with a sharp increase of inflammatory cytokines, leading to cytokine release syndrome (CRS) which was the most common and lethiferous side effect in CAR-T therapy. CAR-T cells co-cultured with target AML cells enhanced secretion of inflammatory cytokine such as IFN-γ, IL-2, and TNF-α. Recent research found that scFv-based FLT3 CAR-T cells released significantly increased IL-2 and IFN-γ when co-cultured with target cells [11], while in our study, IL-2 was slightly increased when co-cultured with FLT3-ITD leukemia cells and nearly not changed when co-cultured with FLT3-WT leukemia cells. Similar to the cell line results, IL-2 was nearly not detectable in the system of CAR-T cells co-cultured with primary FLT3^+^ AML cells. We suppose that this may relate with different recognizing domains of CAR, FLT3L, and scFv and result in different interaction patterns and different types of cytokine profiles released by CAR-T.

CRS and on-target/off-tumor effect are the prominent toxicities of CAR-T therapy. Over the years, improvements in CAR-T cells have been performed to make AML CAR-T therapy more safe and effective. Effective means such as suicide gene, inhibitory CAR, dual-antigen receptor, on-switch CAR, and combination with checkpoint blockade therapy have been studied to control CAR-T cells effectively in vivo [41]. Researchers were also trying to find specific or even unique antigens for CAR-T therapy and avoid irreversible on-target/off-tumor cytotoxicity on normal cells. However, unique antigen expressed only on leukemia cells was scarcely to be found, and on-target/off-tumor toxicity may happen at various degrees. The toxicity is mainly due to the inability of CAR-T cell to distinguish between normal cells and tumor cells. To find one type of recognizing domain which can distinguish between normal cell and tumor cell will have a significant impact on CAR-T therapy. For this goal, we described a new type of CAR-T cell directed to FLT3 using its ligand as recognizing domain, the FLT3L CAR-T cell, and demonstrated that it had potent but diverse antitumor activity to FLT3-WT and FLT3-ITD leukemia cells. FLT3 ligand-based CAR-T might induce activation of proliferation-related signaling of FLT3-WT cells, indicating that FLT3L CAR-T was able to distinguish between FLT3 wild-type cells and FLT3 mutant cells. Besides that, as distinct from scfv-based FLT3 CAR-T, our study showed diverse release of inflammatory cytokine. More importantly, the FLT3L CAR-T had little off-target cytotoxicity on normal HSPCs. In vivo FLT3^+^ leukemia model showed that FLT3L CAR-T could significantly prolong the survival of leukemia beard mice. In conclusion, FLT3 ligand-based CAR-T cells exhibited specific cytotoxicity against FLT3+ leukemia cell and had the ability to distinguish between FLT3 wild-type cells and FLT3-ITD mutant cells, which may be an ideal candidate that specifically target AML with FLT3 mutation.

## Conclusions

FLT3L CAR-T cells exhibited specific cytotoxicity against FLT3^+^ leukemia cell lines and primary AML cells in vitro, particularly FLT3-ITD leukemia cell lines. In vivo treatment of FLT3L CAR-T cells could significantly prolong the survival of FLT3^+^ mouse model. Especially, FLT3L CAR-T cells could promote the phosphorylation of ERK1/2 in FLT3-WT cells but not FLT3-ITD cells, and it had no inhibitory effects on CD34^+^ stem cell colony formation, which indicated its stronger cytotoxic effect on FLT3-ITD leukemia cells and potential in protection of normal HPSCs. In conclusion, FLT3 ligand-based CAR-T cell may be an ideal strategy to target AML with FLT3 mutation.

## Additional file

Additional file 1: **Figure S1.** Flow cytometry analysis of CD45^+^CD33^+^ leukemia cells in peripheral blood of 14 and 7 days before death of leukemia mice. **Figure S2.** FLT3 SFI of three cord blood CD34^+^ HSCs, five FLT3^+^ leukemia cell lines, and leukemia cells of ten AML patients were analyzed by flow cytometry. (PNG 1277 kb)

## Abbreviations

ALL: Acute lymphoblastic leukemia
AML: Acute myeloid leukemia
BD: Binding domain
BMMC: Bone marrow mononuclear cell
CRS: Cytokine release syndrome
FLT3: Fms-like tyrosine kinase
GMP: Granulocyte/macrophage progenitor
HPSC: Hematopoietic progenitor stem cells
HSCT: Hematopoietic stem cell transplantation
IFN-γ: Interferon-gamma
IL-2: Interleukin-2
ITD: Internal tandem duplications
MHC: Major histocompatibility complex
RT: Room temperature
scFv: Single-chain variable fragment
SFI: Specific fluorescence indices
TAA: Tumor-associated antigen
Tcm: Central memory T cell
Tem: Effector memory T cell
Temra: Terminally differentiated effector memory T cell
TKD: Tyrosine kinase domain
TNF-α: Tumor necrosis factor-alpha
WT: Wild type
